# Hard and soft tissue contour changes following simultaneous guided bone regeneration at single peri-implant dehiscence defects using either resorbable or non-resorbable membranes: a 6-month secondary analysis of a randomized controlled trial

**DOI:** 10.1007/s00784-025-06322-4

**Published:** 2025-04-08

**Authors:** Franz J. Strauss, David Schneider, Ronald E. Jung, Riccardo Kraus, Daniel S. Thoma, Nadja Naenni

**Affiliations:** 1 Clinic of Reconstructive Dentistry, Center of Dental Medicine, University of Zurich, Zurich, Switzerland; 2 Faculty of Health Sciences, Universidad Autonoma de Chile, Providencia, Chile; 3 Department of Periodontology, Research Institute for Periodontal Regeneration, Yonsei University College of Dentistry, Seoul, Republic of Korea

**Keywords:** Dental implants, Bone regeneration, Bone generation, Alveolar ridge augmentation

## Abstract

**Objectives:**

To compare radiographic and profilometric outcomes 6 months after simultaneous lateral guided bone regeneration (GBR) at single peri-implant dehiscence defects in the anterior region using either resorbable or non-resorbable membranes.

**Materials and methods:**

In 27 patients with a single tooth gap in the anterior region (second premolar to second premolar in the maxilla) a dental implant was placed. Following implant placement GBR was performed at the buccal aspect using randomly either a resorbable collagen membrane (RES) or a non-resorbable titanium-reinforced ePTFE membrane (N-RES). Radiographic (cone-beam computed tomography; CBCT) measurements were performed to assess the buccal bone thickness immediately after the implant placement with simultaneous GBR (baseline) and 6 months later. Buccal soft tissue thickness was assessed by superimposing surface scans taken at baseline and again 6 months later.

**Results:**

A total of 25 datasets could be assessed for the bone dimensions (*n* = 12, RES; *n* = 13, N-RES) and 14 datasets for profilometric changes (*n* = 7, RES; *n* = 7, N-RES). Group RES showed a significant mean reduction in buccal bone between baseline and 6 months of 0.8 ± 0.4 mm (*p* = 0.004). The respective mean reduction for group N-RES amounted to 0.1 ± 0.4 mm (*p* = 0.581). When comparing the buccal bone changes between both group over time, group RES exhibited greater reduction in comparison to group N-RES (intergroup *p* = 0.017). Profilometric analyses showed a non-significant trend towards soft tissue gain in group RES 0.6 ± 0.7 mm (*p* = 0.125). Conversely, N-RES group revealed stability, with a mean change of 0.0 ± 0.3 mm (*p* = 1.000).

**Conclusions:**

GBR using non-resorbable membranes seems to provide greater dimensional stability of augmented bone at 6 months re-entry and before implant loading compared to resorbable membranes. The lack of differences in the profilometric outcomes and contour changes may be explained by a partial compensation through an increase in soft tissue thickness with resorbable membranes.

**Clinical relevance:**

GBR using non-resorbable membranes may offer greater dimensional stability of augmented bone compared to resorbable membranes. However, these potential benefits may be offset by a compensatory increase in soft tissue thickness when using resorbable membranes.

**Supplementary Information:**

The online version contains supplementary material available at 10.1007/s00784-025-06322-4.

## Introduction

Dental implant placement is often combined with guided bone regeneration (GBR) procedures to regenerate missing hard tissue or compensate for volume loss resulting from tooth extraction [1–4]. The trend towards backward planning in implant dentistry has resulted in placing dental implants in positions that align with prosthetic needs, often causing small buccal peri-implant dehiscences. Treating these dehiscences with GBR can prevent vertical bone loss in about half of the cases [5], minimise mucosal recession [6], and prevent biological complications such as bleeding on probing and deeper probing pocket depths [7]. Not treating these defects can negatively affect aesthetic outcomes and patient satisfaction [8]. Additionally, implants with exposed threads are more prone to peri-implantitis [9].

Various materials for GBR have been evaluated, with many yielding positive clinical results [10–16]. In general, GBR technique employs a membrane to prevent soft tissue ingrowth into the defect space. However, successful bone growth relies on the membrane maintaining its structural integrity without collapsing [17]. To achieve this, one approach involves reinforcing the membrane by integrating titanium into expanded polytetrafluoroethylene (ePTFE) membranes. Despite the high clinical success rates of titanium-reinforced (ePTFE) membranes [18–20], their application poses clinical challenges [21]. For example, non-resorbable ePTFE membranes require an additional surgical procedure for removal. In addition, they are susceptible to bacterial colonization and infection, potentially necessitating premature removal and negatively impacting augmented bone volume [22–26].

While clinical studies report favorable outcomes with these membranes, incomplete bone regeneration has been observed, leading to biologic and aesthetic compromises [27–29]. Notably, there is limited comparative data on dimensional alterations after implant placement with simultaneous GBR procedures performed with resorbable and non-resorbable membranes [30–32]. This comparison is crucial given the widespread use of GBR in daily practice. This warrants an assessment of their respective performance in terms of hard and soft tissue contour changes for informed decision making. To date, only few studies have investigated the changes in contour and morphology of the labial peri-implant tissues when using resorbable and non-resorbable membranes [33–36].

Based on the inherent space-making stability of non-resorbable membranes, it is reasonable to hypothesise that augmented bone using non-resorbable membranes will have a higher dimensional stability compared to augmented bone using resorbable membranes. Therefore, the aim of the present study was to compare radiographic (bone dimensions) and profilometric outcomes after guided bone regeneration (GBR) at single implant sites in the anterior region using either a resorbable or a non-resorbable membrane.

## Methods

### Study design

The present secondary study reports the results of a parallel-group randomized controlled trial assessing resorbable or non-resorbable membranes for guided bone regeneration at peri-implant bone defects [37]. The study protocol received the approval from the local ethical committee from the canton Zurich, Switzerland (Ref. KEK–Zh-Nr. 2010–0051/5) and adheres to the principles of the Declaration of Helsinki of 1975, revised in Fortaleza in 2013. The study design and reporting conform to the CONSORT statement guidelines (http://www.consort-statement.org/). The study was not registered in a trial database since it started in 2010. All interventions were performed by experienced clinicians at the Clinic of Reconstructive Dentistry, Center for Dental Medicine, University of Zurich, Switzerland. Fifty-one patients were screened presenting with a single tooth gap in the anterior region of either jaw requiring implant treatment as previously described in the original publication [37]. In brief, implants had to present with a buccal dehiscence defect of at least 3 mm, requiring simultaneous guided bone regeneration (GBR). The choice of membrane was made according to a computer-generated randomization list. In brief, patients were randomly assigned to the following groups:

1. **RES:** Simultaneous lateral bone augmentation using demineralized bovine bone mineral (DBBM, Bio-Oss granules, particle size 0.25–1.0 mm; Geistlich Pharma AG, Wolhusen, Switzerland) covered with a collagen membrane (CM, Bio-Gide® Geistlich Pharma).
2. **N-RES:** Simultaneous lateral bone augmentation using demineralized bovine bone mineral (DBBM, Bio-Oss granules, particle size 0.25–1.0 mm; Geistlich Pharma AG, Wolhusen, Switzerland) covered with a non-resorbable, titanium reinforced ePTFE-membrane (Gore-Tex®, W.L. Gore & Assoc., Flagstaff, Arizona, USA).

All patients were thoroughly informed in oral and written form about the study procedures. Patients had to fulfil the following inclusion criteria:

- Minimum age of 18 years
- General health showing no contraindications regarding implant treatment
- Periodontal health with probing depths < 4 mm [38]
- Good oral hygiene (full mouth plaque scores < 25% [39]
- Full mouth bleeding on probing scores < 25% [40].
- If necessary, patients were enrolled in a structured oral hygiene program until the respective periodontal condition was reached.
- Tooth extraction at least 6 weeks prior to implant placement.
- Expected dehiscence defect after implant placement requiring GBR procedure.
- Light smokers, fewer than 10 cigarettes per day.

If no GBR procedure was necessary following implant placement, patients were ineligible for the study. A total of 27 patients who met this criterion and provided written consent were included in the study.

### Surgical procedure & implant placement

Prior to surgery, patients received analgesics (Mefenacid 500 mg, Mepha Pharma AG, Basel, Switzerland) and had to rinse with chlorhexidine 0.2% for one minute. After local anesthesia (Articaine hydrochloride, Rudocaine forte®, Streuli, Switzerland), an intrasulcular incision and one vertical releasing incision at the distal aspect of the implant site was made. Subsequently, a full-thickness flap was raised. The implant bed was prepared according to the manufacturer’s recommendations and a titanium implant (OsseospeedS, AstraTech, Mölndal, Sweden) was inserted in a prosthetically driven position. All implants obtained primary stability.

Clinical measurements of the bone anatomy (dehiscence, intrabony defect width and depth) were assessed at the time of surgery and again at the re-entry procedure 6 months later. Only patients exhibiting dehiscence bone defects at the buccal aspect were included in the study.

A study monitor revealed the group allocation to the surgeon only after the implant was placed. Bone augmentation was performed in both groups using demineralized bovine bone mineral (DBBM, Bio-Oss granules, particle size 0.25–1.0 mm; Geistlich Pharma AG, Wolhusen, Switzerland). In group RES, the collagen membrane was trimmed and applied to cover the DBBM. In order to avoid displacement of membrane and/or DBBM resorbable pins made of polylactic acid (Resor Pins; Geistlich AG, Wolhusen, Switzerland) were used to fix the membrane to the buccal bone in the apical region. In group N-RES, the ePTFE-membrane was also trimmed and shaped and adapted to the defect site. The membrane was fixed bucally using two non-resorbable titanium pins (Frios®, Friadent GmbH, Mannheim, Germany) apically. Both membranes overlapped the borders of the augmented defect site by at least 2 mm. Careful consideration was given to ensure that the non-resorbable membrane would not come into contact with the adjacent teeth to prevent potential complications. At the buccal aspect the membranes were placed under the full-thickness flap. Buccal flap mobilization was achieved by means of a periosteal releasing incision and the site was sutured with non-resorbable sutures (Gore-Tex suture; Gore, Flagstaff, AZ, USA) aiming at healing by primary intention.

After surgery, patients were advised to refrain from mechanical plaque removal in the area for 7–10 days and to rinse with a 0.2% chlorhexidine solution (Kantonsapotheke, Zurich, Switzerland) twice a day. Antibiotics (Amoxicillin 750 mg, Sandoz Pharmaceuticals, Risch, Switzerland) were prescribed 3x/d for 5 days and analgesics (Mefenacid 500 mg, Mepha Pharma AG, Basel, Switzerland) according to individual needs. Sutures were removed 7–10 days after surgery.

### Follow-up

Immediately after completion of the surgery, a cone beam computed tomogram (CBCT) (KaVo Dental GmbH, Biberach, Germany; 120 kV, 5 mA, 0.25 mm voxel size) of the treated jaw including the area with the implant and the GBR procedure was taken. This digital radiographic data set served as the baseline for bone dimension measurements (BLb).

Four weeks post implant placement a silicone impression was taken (President, Coltène/Whaledent, Altstaetten, Switzerland) and cast models were fabricated and later digitized using an optical lab scanner (Iscan D101, Imetric GmbH, Courgenay, Switzerland). This surface scan data set served as the baseline for the soft tissue contour measurements (BLs).The obtained Standard Tessellation Language (STL) files and the radiographic baseline data (Digital Imaging and Communication in Medicine, (DICOM)) were superimposed using software (SMOP, Swissmeda, Zurich, Switzerland) applying a best-fit algorithm using the existing dentition as the reference structure.

Six months after the surgery, and before re-entry, another silicone impression was taken. A cast model was subsequently created and digitized, using the previously described method. At this stage, mucoperiosteal flaps were elevated at all sites allowing for clinical measurements of bone morphology. Additionally, a second CBCT was taken at this time point. In group RES the CBCT was taken before flap elevation, whereas in group N-RES the membranes were removed prior to the CBCT scan to prevent artifacts. The digital surface data and the radiographic dataset were then superimposed once more using the same software.

### Data analysis

**Fig. 1:**
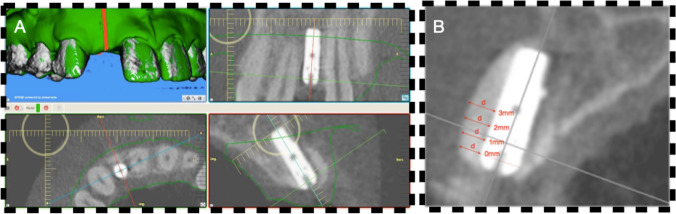
Illustration of bone and soft tissue contour measurements. **A** The top left window displays a screenshot from the measurement software, featuring a CBCT image with a superimposed STL file indicating the implant site (#12). The green surface represents the digitally scanned cast model. A red line demarcates the bucco-oral section in the bottom left window. Sections along (top right) and perpendicular (bottom right) to the implant are shown in the right windows. The dimensional measurements were conducted on the section displayed in the bottom right window, with bone dimensions measured on the CBCT image (grey) and soft tissue dimensions on the superimposed STL file (green outline). **B** Measurements of the buccal bone dimensions perpendicular to the implant axis at the level of the implant shoulder (0 mm) and at increments of 1, 2 and 3 mm apical to the shoulder

**Fig. 2:**
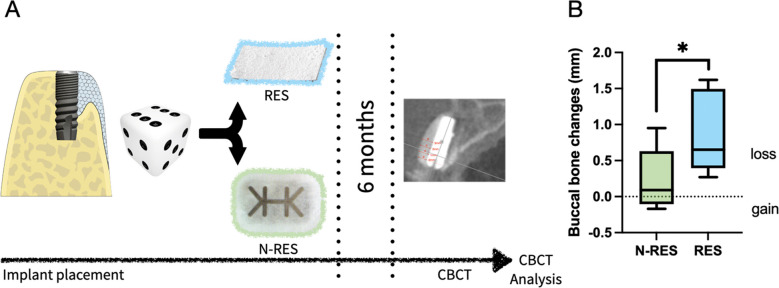
**A** Study timeline. **B** Buccal bone changes based on CBCT in both treatment groups. Differences were calculated using Wilcoxon Mann–Whitney U-test. *indicates p-value < 0.05

The data from both time points (baseline and 6-months follow-up) were analyzed using the above-mentioned software program (Figs. 1 and 2). The following measurements were made at baseline and at 6 months:

- Buccal bone thickness in a bucco-oral dimension perpendicular to the implant axis at the level of the implant shoulder (0 mm) and at 1, 2 and 3 mm below.
- Buccal soft tissue thickness in a bucco-oral dimension perpendicular to the implant axis at the level of the implant shoulder (0 mm) and at 1, 2 and 3 mm below.

### Sample size

Information on the sample size calculation is provided in the original publication, based on vertical defect fill [37]). The calculation was performed using G*Power (Faul, F., Erdfelder, E., Buchner, A., & Lang, A.-G., 2009) via a two-independent-sample means test (two-sided) with data from a previous study (Jung et al., 2009). Assuming a clinically relevant difference of 1.0 mm in vertical defect fill after GBR at the 6-month re-entry and a common standard deviation of 1.0 mm, a significance level of α = 0.05, and 80% power, 34 patients were needed to detect a significant difference in vertical defect fill.

### Statistical analysis

Descriptive statistics were calculated including mean, median, SD and upper/lower quartile (Q1, Q3) for continuous variables. Analysis of statistical significance encompassed non-parametric methods, because of the small samples and the non-symmetric data distributions. Hence, medians with quartiles were provided. Significance was determined using the Wilcoxon Mann–Whitney U-test for the comparison of the two treatments. The Sign test was applied for the changes of the measurements, because of the non-symmetry of the data distribution. The p-values were based on exact derivation. The level of significance was set at *p* < 0.05. No correction was applied for the multiple testing.

## Results

### Study sample

Out of 27 patients included in the study, 25 datasets could be assessed for the bone dimensions (*n* = 12, RES; *n* = 13, N-RES) (Fig. 2A). Patient demographics including defect dimensions at baseline are displayed in Table 1. Data of fourteen patients (*n* = 7, RES; *n* = 7, N-RES) was suitable for the analyses of both bone dimensions (CBCT) and the soft tissue dimensions (surface scan). In the remaining cases data could not be utilized due to pronounced artifacts (impression/cast model quality), which did not allow accurate assessments.

**Table 1:** Participants baseline characteristics

	Group RES	Group N-RES
N (%)	13	14
Age, mean (SD)	50.5 (19.7)	54.3 (16.7)
Gender		
Female	6	8
Male	7	6
Implant site, N		
Incisors	3	9
Premolars	13	2
Defect dimensions (mm)		
Width (mean (SD); median)	3.0 (0.1); 3	3.1 (0.3); 3
Depth (mean (SD); median)	0.9 (0.2); 1	1.0 (0.2); 0
Vertical defect (mean (SD); median)	4.0 (2.0); 4	2.3 (2.0); 2.5

### Buccal bone thickness

The overall mean buccal bone thickness amounted to 2.8 ± 0.6 in group RES (Table 2) and 2.4 ± 0.7 in group N-RES (Table 3). A significant reduction in buccal bone thickness between baseline and 6 months follow-up was observed in group RES at all levels (intragroup *p* < 0.05) (Table 4). The mean reduction in buccal bone thickness at all four levels amounted to 0.8 mm. In contrast, the corresponding mean reduction in the N-RES group at 6 months was 0.1 mm, without significant differences compared to baseline (intragroup p > 0.05) regardless of the level measured (Table 3). When comparing the buccal bone changes between both group over time, group RES exhibited greater reduction in comparison to group N-RES (intergroup *p* = 0.017) (Fig. 2B).

**Table 2:** Overall buccal bone dimensions (mm) and dimensions at the different levels (group RES, *N* = 12)

RES	Mean	SD	Q1	Median	Q3	p value
Overall buccal bone at baseline	2.8	0.6	2.5	3.0	3.2	
Overall buccal bone at 6 months	1.9	0.8	1.4	1.9	2.7	
Overall buccal bone difference	0.8	0.4	0.5	0.6	1.3	**0.004**
Shoulder baseline	2.0	0.7	1.7	2.0	2.5	
Shoulder 6 months	1.2	0.9	0.2	1.4	1.9	
Bone difference	0.7	0.7	0.3	0.6	1.4	**0.006**
1 mm baseline	2.6	0.6	2.4	2.8	3.0	
1 mm 6 months	1.6	1.0	1.0	1.8	2.5	
Bone difference	0.9	0.6	0.5	0.7	1.2	**0.0005**
2 mm baseline	3.1	0.8	2.7	3.3	3.6	
2 mm 6 months	2.2	0.8	1.7	2.2	3.0	
Bone difference	0.9	0.4	0.4	1.0	1.3	**0.0005**
3 mm baseline	3.3	0.8	2.7	3.5	3.9	
3 mm 6 months	2.5	0.8	1.9	2.5	3.2	
Bone difference	0.7	0.5	0.3	0.8	1.1	**0.006**

*RES* resorbable membrane, *N-RES* non-resorbable (ePTFE) membrane

**Table 3:** Buccal bone dimensions (mm) and dimensions at the different levels (group N-RES, *N* = 13)

N-RES	Mean	SD	Q1	Median	Q3	p value
Buccal bone at baseline	2.4	0.7	2.2	2.5	2.8	
Buccal bone at 6 months	2.2	0.8	1.9	2.3	0.3	
Bone difference	0.1	0.3	−0.0	−0.0	0.3	**0.581**
Shoulder baseline	2.1	1.0	1.8	2.1	2.6	
Shoulder 6 months	1.9	1.1	1.7	2.1	2.3	
Bone difference	0.1	0.3	−0.0	0.1	0.5	**0.387**
1 mm baseline	2.4	0.8	2.2	2.5	2.7	
1 mm 6 months	2.2	0.9	1.9	2.4	2.6	
Bone difference	0.1	0.4	−0.0	0.1	0.3	**0.387**
2 mm baseline	2.5	0.6	2.2	2.5	2.9	
2 mm 6 months	2.3	0.8	1.9	2.3	2.8	
Bone difference	0.2	0.4	−0.0	0.1	0.3	**0.581**
3 mm baseline	2.5	0.6	2.1	2.5	3.0	
3 mm 6 months	2.4	0.5	2.2	2.4	2.9	
Bone difference	0.1	0.3	−0.0	0.0	0.1	**1.000**

*RES* resorbable membrane, *N-RES* non-resorbable (ePTFE) membrane

**Table 4:** Overall soft tissue dimensions (mm) and dimensions at the different levels (group RES, *N* = 7)

RES	Mean	SD	Q1	Median	Q3	p value
Overall soft tissue at baseline	1.6	0.9	0.9	1.5	1.8	
Overall soft tissue At 6 months	2.2	0.9	1.4	1.9	2.8	
Overall soft tissue difference	0.6	0.6	0.8	0.4	0.2	**0.125**
Shoulder baseline	1.8	0.8	1.1	1.5	2.3	
Shoulder 6 months	2.4	1.0	1.9	2.2	3.4	
Soft tissue difference	0.5	1.1	1.1	0.7	0.3	**0.453**
1 mm baseline	1.6	0.9	0.9	1.5	1.8	
1 mm 6 months	2.3	0.9	1.7	2.0	3.4	
Soft tissue difference	0.6	0.9	1.1	0.4	0.2	**0.125**
2 mm baseline	1.4	0.9	0.8	1.0	1.5	
2 mm 6 months	2.1	1.1	1.5	1.6	2.6	
Soft tissue difference	0.7	0.5	1.2	0.6	0.4	**0.031**
3 mm baseline	1.5	1.0	0.8	1.4	1.6	
3 mm 6 months	1.9	0.9	1.4	2.0	2.4	
Soft tissue difference	0.4	0.5	0.9	0.4	0.2	**0.453**

*RE* resorbable membrane, *N-RES* non-resorbable (ePTFE) membrane

### Soft tissue thickness

The overall mean buccal soft tissue thickness at baseline amounted to 1.6 ± 0.9 in group RES (Table 4) and 1.9 ± 0.4 in group N-RES (Table 5). At 6 months, the overall mean buccal soft tissue thickness amounted to 2.2 ± 0.9 in group (RES) indicating a gain of ≈ 0.6 mm (intragroup *p* = 0.125). Conversely, in the group N-RES the overall mean buccal soft tissue thickness remained unchanged (intragroup *p* = 1.000), amounting to 1.9 ± 0.5. When comparing the changes in buccal soft tissue thickness between both groups over time, group RES showed a trend toward gains in comparison to group N-RES (intergroup *p* = 0.097).

**Table 5:** Overall soft tissue dimensions (mm) and dimensions at the different levels (group N-RES, *N* = 7)

N-RES	Mean	SD	Q1	Median	Q3	p value
Overall soft tissue at baseline	1.9	0.4	1.6	2.0	2.2	
Overall soft tissue At 6 months	1.9	0.5	1.4	2.0	2.3	
Overall soft tissue difference	0.0	0.3	0.3	0.0	0.2	**1.000**
Shoulder baseline	1.7	0.7	1.0	1.6	2.2	
Shoulder 6 months	1.9	0.6	1.5	1.6	2.1	
Soft tissue difference	0.1	0.4	0.6	0.2	0.0	**0.453**
1 mm baseline	1.8	0.5	1.4	1.9	2.0	
1 mm 6 months	1.9	0.6	1.2	1.8	2.4	
Soft tissue difference	0.0	0.4	0.4	0.1	0.2	**0.453**
2 mm baseline	2.0	0.4	1.7	2.2	2.3	
2 mm 6 months	2.0	0.6	1.4	2.1	2.7	
Soft tissue difference	0.0	0.6	−0.5	0.1	0.3	**0.453**
3 mm baseline	2.2	0.4	2.0	2.2	2.6	
3 mm 6 months	1.9	0.5	1.4	1.8	2.4	
Soft tissue difference	0.2	0.2	0.0	0.2	0.5	**0.125**

*RE* resorbable membrane, *N-RES* non-resorbable (ePTFE) membrane

### Contour changes

When superimposing CBCT with STL data, group RES showed an overall mean contour loss of 0.2 ± 0.4 at 6 months follow-up. Similarly, group N-RES exhibited an overall mean contour loss of 0.1 ± 0.3 at 6 months.

## Discussion

The present RCT comparing radiographic and profilometric outcomes after guided bone regeneration (GBR) at single implants using either resorbable or non-resorbable membranes revealed at 6 months follow-up:

i. resorbable membranes show significantly greater buccal bone reduction compared to non-resorbable membranes; however, this higher reduction appears to be offset by an increase in soft tissue thickness.
ii. A trend toward soft tissue gain with the use of resorbable membranes, whereas soft tissue thickness seems to remain stable when using non-resorbable membranes.
iii. A stability of the overall buccal contour throughout the observation period in both groups, suggesting a compensatory mechanism in soft tissue thickness.

The augmented bone thickness exhibited significantly greater stability when titanium-reinforced non-resorbable membrane was used. Buccal bone loss predominantly occurred in group RES, where a resorbable collagen membrane had been used. These radiographic findings are in line with previously published data of the clinical parameters at re-entry [37] despite the challenges in detecting very thin bony layers on CBCT images [4, 41, 42].

Some authors have published long-term data demonstrating that even a thin or radiographically undetectable buccal bone plate may not compromise the aesthetic outcome [31, 43]. The loss of buccal bone seems, at least in part, to be compensated by an increase in soft tissue thickness, contributing to favourable aesthetic outcomes. This phenomenon parallels observations in post-extraction sockets [44, 45]. In addition, the current findings suggest an even more pronounced increase in soft tissue thickness in the group that showed a greater decrease in bone thickness. Hence, these observed tissue dynamics led to a relatively stable overall outer contour throughout the 6-month follow-up in both groups.

The mean change in soft tissue over the 6-month follow-up, measured between baseline and re-entry, showed non-significant changes within (≈0.6 mm; RES);(0.0 mm; N-RES) and between the groups. No additional soft tissue augmentation procedures and prosthetic deliveries occurred during this period. Consequently, significant changes in soft tissue thickness were not anticipated during this healing phase. Unlike scenarios with an implant supported crown, which could lead to apical and buccal a shift in soft tissue [46], no such pressure-induced increase was expected due to the absence of treatment. Nevertheless, within group RES there was a notable increase of about 0.6 mm in soft tissue thickness over time. The potential influence or promotion of this increase by the rather fast resorption rate and potential tissue integration of the membrane remains speculative. In contrast, no changes were found in group N-RES. The reasons for these differences in soft tissue changes between the groups remain unclear.

The results of the present study indicate that the mean hard tissue loss observed in group RES from baseline to 6 months (0.8 mm) was offset by a simultaneous increase in soft tissue (≈0.6 mm). This compensatory phenomenon could not be observed in group N-RES. In this group, measurements revealed stability with minimal mean changes in bone (≈0.1 mm) and in soft tissues (≈0.0 mm). Overall, these changes led to a minor reduction in the outer contour, with a decrease of approximately 0.2 mm in group RES and 0.1 mm in group N-RES. These observations are in line with previous clinical studies [31, 43] where even in the absence of detectable buccal bone on CBCT scans, the long-term aesthetic outcomes were not adversely affected. Thus, it seems that a potential loss in hard tissue thickness can be compensated by an increase in soft tissue [45, 47], a trend corroborated by the results of this study.

The present study has several limitations. Firstly, only 14 out of the 27 patients could be included in the simultaneous analysis of bone and soft tissue changes, which may introduce some bias. The small sample size made it impractical to adjust for other potential covariates [48] such as vertical defect dimensions. However, despite the limited sample size and statistical power, significant differences were observed. These findings may serve as valuable data for future systematic reviews and meta-analyses aimed at calculating pooled estimates [49] comparing resorbable and non-resorbable membranes in GBR procedures. Additionally, they could significantly contribute to Individual Participant Data (IPD) meta-analyses of RCTs, which provide greater statistical power and more robust results compared to conventional meta-analyses based on aggregate data [50]. Secondly, four dehiscences were observed in the RES group and two in the N-RES group, potentially influencing the results. Although resorbable membranes have a capacity for spontaneous healing after exposure [51], a recent meta-analysis indicated that membrane exposure—whether involving non-resorbable or resorbable membranes—can negatively impact bone regeneration at peri-implant sites [52]. Thirdly, while all patients underwent tooth extraction 6 weeks prior to implant placement, the procedures were not performed in the same week since some patients were referred to our clinic with the tooth already extracted. Therefore, a potential influence of the catabolic events that occur post-extraction on the present findings cannot be dismissed despite the randomisation process. Fourthly, the amount of bone grafting was not quantified and was left to the discretion of the practitioner based on the clinical situation, which could introduced variability in the outcomes. Finally, it remains unclear whether the fixation material (polylactic acid resorbable pins and titanium pins), the surgical trauma (e.g. flap elevation) and the thickness of the non-resorbable membrane may have influenced the results. Larger RCTs are warranted to confirm these findings.

## Conclusions

GBR using non-resorbable membranes seems to provide greater dimensional stability of augmented bone at 6 months re-entry and before implant loading compared to resorbable membranes The lack of differences in the profilometric outcomes and contour changes may be explained by a partial compensation through an increase in soft tissue thickness with resorbable membranes.

## Supplementary Information

Below is the link to the electronic supplementary material.

Supplementary file1 (DOC 219 KB)

## Data Availability

No datasets were generated or analysed during the current study.
