# Correction to: Hard and soft tissue contour changes following simultaneous guided bone regeneration at single peri‑implant dehiscence defects using either resorbable or non-resorbable membranes: a 6-month secondary analysis of a randomized controlled trial

**DOI:** 10.1007/s00784-026-06771-5

**Published:** 2026-02-05

**Authors:** Franz J. Strauss, David Schneider, Ronald E. Jung, Riccardo Kraus, Daniel S. Thoma, Nadja Naenni

**Affiliations:** 1 Clinic of Reconstructive Dentistry, Center of Dental Medicine, University of Zurich, Zurich, Switzerland; 2 Faculty of Health Sciences, Universidad Autonoma de Chile, Providencia, Chile; 3 Department of Periodontology, Research Institute for Periodontal Regeneration, Yonsei University College of Dentistry, Seoul, Republic of Korea

**Correction to: Clin Oral Invest**

10.1007/s00784-025-06322-4

In the Materials section, within the resorbable collagen membrane (RES) group, the material was incorrectly reported as **“Geistlich Mucograft**®** Seal” (Geistlich Pharma)**. The correct material used should be **“Bio-Gide**® **(Geistlich Pharma)”**.

Although this involves a change of only one term, it is clinically and scientifically relevant, as these are different materials with distinct indications.

The original article has been corrected.

